# Estimating the Number of People Eating Biofortified Foods On-Farm and from Markets: a Detailed Methodology and Tool

**DOI:** 10.1016/j.cdnut.2026.107653

**Published:** 2026-02-05

**Authors:** Richard Alioma, Rita Wegmüller, Bho Mudyahoto, James P Wirth, Wolfgang Pfeiffer, Munawar Hussain, Erick Boy

**Affiliations:** 1 HarvestPlus, International Food Policy Research Institute, Washington, D.C., United States; 2 GroundWork, Fläsch, Switzerland; 3 Alliance Bioversity & International Center for Tropical Agriculture, Rome, Italy

**Keywords:** biofortification, biofortified foods, reach, coverage, methodology, program management

## Abstract

**Background:**

Biofortification is a cost-effective and scalable approach to reducing micronutrient deficiencies. Currently, there are scant data detailing the number (reach) and proportion (coverage) of individuals consuming biofortified foods, which is a key limitation for policymakers.

**Objectives:**

This study aimed to develop a method to estimate the reach and coverage of biofortified foods using primary and secondary data sources.

**Methods:**

We used data from 2023 to estimate the reach and coverage of zinc biofortified rice in Bangladesh and wheat in Pakistan, and vitamin A maize and cassava in Nigeria. Our calculation is divided into 5 phases: *1*) seed availability, *2*) agricultural production, *3*) on-farm consumption, *4*) off-farm consumption, and *5*) overall national level reach and coverage. Phase 4 includes 2 consumption scenarios: full replacement and half replacement, where biofortified foods, respectively, account for 100% or 50% of the food crop per capita consumption.

**Results:**

In 2023, ∼13–16 million people (8%–9% of the population) consumed biofortified rice in Bangladesh. In Pakistan, between 97 and 173 million people consumed biofortified wheat (39%–70% of the population). In Nigeria, biofortified maize was consumed by 42–66 million people (18%–29% of the population) and biofortified cassava by 25–38 million people (11%–17% of the population).

**Conclusions:**

Our method estimates on-farm and off-farm reach and the reach/coverage of biofortified foods with visible and invisible traits. Because there is insufficient primary or secondary data describing the intake of biofortified foods, we estimated a range for off-farm reach. We estimate that in 2023, between 177 and 293 million people consumed the 4 biofortified crops explored in this analysis. This approach can be used to estimate the reach and coverage of other biofortified crops in other countries. More information about the consumption of these foods is usually needed to improve the accuracy of national reach and coverage estimates.

## Introduction

Micronutrient deficiencies affect billions of people worldwide, with a higher prevalence in low- and middle-income countries (LMICs), particularly in sub-Saharan Africa and South Asia [1,2]. Such deficiencies negatively affect child growth, physical and mental development [3], and increase the risk for infections by compromising the immune system [4]. Most recent estimates suggest that over half of preschool-aged children and over two-thirds of women of reproductive age worldwide suffer from ≥1 micronutrient deficiency [1].

Biofortification, or the breeding of staple crop varieties with higher concentrations of vitamins and minerals, is an evidence-based, cost-effective, and scalable approach to address micronutrient deficiencies. It is particularly suitable for rural, low-income settings where market access is limited, and farming households primarily consume what they grow [5,6]. Multiple efficacy trials [[7], [8], [9], [10]] and effectiveness studies [11,12] have demonstrated the positive effects of biofortified food consumption on micronutrient status and health outcomes. Several narrative reviews have also summarized the health impact and socioeconomic feasibility of biofortified foods [5,13,14].

Large-scale promotion and delivery of biofortified crop varieties began more than a decade ago, and rapid scaling is already happening in several countries. By 2024, >450 biofortified varieties of 11 staple crops (e.g., beans, cassava, cowpeas, lentils, maize, orange sweet potatoes, pearl millet, banana/plantain, rice, sorghum, and wheat) had been released in 41 LMICs in Africa, Asia, and Latin America [5,15].

As biofortification programs mature and reach scaling phase, policymakers and development partners require regularly updated data on *1*) the number of individuals consuming/accessing biofortified foods (reach), and *2*) the proportion of the population consuming biofortified foods (coverage). The terms “reach” and “coverage” have been commonly used when describing the performance of food fortification programs [16]. Although multiple coverage indicators related to biofortification programs have been identified by Petry et al. [17], “reach” has been previously described as the “number of people who consume biofortified foods” in an indicator framework to monitor biofortification programs by Friesen et al. [18]. As biofortification programs initially targeted farming households that use most of the harvested biofortified crop for their own consumption, estimates of the “on-farm” reach of biofortified foods were made by multiplying the number of households growing biofortified foods by the mean household size [19]. This approach does not account for the surplus biofortified food sold and the people who eat biofortified foods obtained from the market (heretofore referred to as “off-farm” reach).

A questionnaire tool for use in household surveys was developed and tested by Petry et al. [17] to assess the coverage of biofortified foods. This tool was able to measure the coverage of biofortified foods with visually recognizable traits (e.g., orange color in vitamin A sweet potato/cassava/maize), but it was suboptimal at estimating the coverage of biofortified foods without visible traits (i.e., high iron beans), because nonfarming respondents could not accurately identify the foods made from such biofortified varieties. More recently, 2 separate publications established a set of biofortification indicators that could be used to monitor the commercialization of biofortified foods [19,20]. These publications describe many indicators, including a calculation of reach based on annual crop production, where the reach of biofortified foods among farming households was calculated by multiplying the number of households growing biofortified foods by the mean farming household size, and “off-farm” reach was calculated by estimating the total quantity of biofortified food available on the market and dividing this quantity by the estimated mean per capita daily intake of the food crop. Our paper refines the approach presented in the above publications and presents in detail the various calculations and data sources utilized by HarvestPlus to assess the performance of its programs.

Using agricultural production and consumption data is the most systematic and reproducible approach to estimate the reach and coverage of biofortified foods, because other methods are confounded by the visual imperceptibility of some biofortified foods (e.g., iron or zinc biofortified beans, pearl millet, maize, rice, and wheat), and the mixing of biofortified and non-biofortified products during harvesting and postharvest [19].

The purpose of this paper is to document the data and equations that comprise the methodology for estimating the reach and coverage of biofortified foods (on-farm and off-farm), so that policymakers, development partners, and researchers have a replicable approach to estimate the reach and coverage of biofortified foods by both farming and nonfarming households. We envision that accurate reach and coverage estimates can improve the management and performance of large-scale nutrition programs and can also be used to estimate the extent to which biofortification programs are contributing to the reduction and prevention of micronutrient deficiencies. Although biofortification programs are monitored using a range of indicators, this manuscript focuses on reach and coverage because these indicators are widely used, comparable across contexts, and well suited to tracking program scale and year-to-year performance.

In this paper, we applied this methodology to 4 crops produced in 3 different countries (zinc biofortified rice in Bangladesh, zinc biofortified wheat in Pakistan, and vitamin A biofortified cassava and maize in Nigeria). We use data for 2023 to illustrate the various calculations and results.

## Methods

**FIGURE 1:**
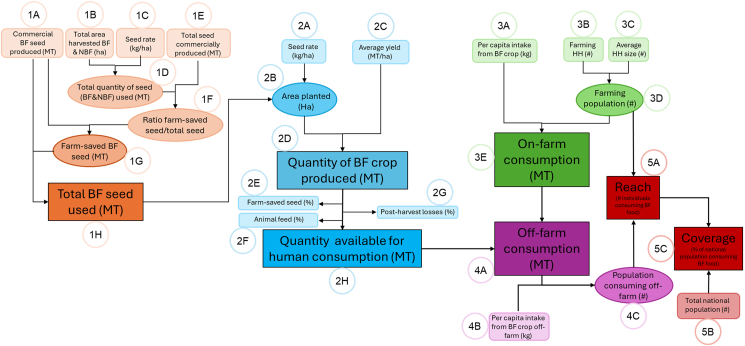
Calculation flow diagram. BF, biofortified; NBF, non-biofortified.

To calculate the reach and coverage of biofortified foods, we developed a calculation comprising 5 phases (Figure 1): *1*) seed availability, *2*) agricultural production, *3*) on-farm consumption, *4*) off-farm consumption, and *5*) overall national level reach/coverage. This method is used by HarvestPlus in the countries it operates to estimate its reach on an annual basis. Figure 1 is a generic illustration of the phases and steps used to calculate reach/coverage.

Each phase in the calculation process requires either the use of primary or secondary data (or both) estimates or an estimate produced by a previous phase. Primary data are defined as data collected by HarvestPlus, and secondary data are defined as publicly available data and estimates (e.g., metrics from FAOSTAT). The small circles displayed in Figure 1 include references to specific data inputs or parameters (e.g., 1B = phase 1, parameter B) that are described in greater detail in Table 1 [[21], [22], [23], [24], [25], [26], [27], [28], [29], [30], [31], [32], [33]]. Input data from 2023 are used for all country-crop combinations in this manuscript. Supplementary Material A provides complete calculation worksheets for the 4 country-crop combinations examined, including the equations and underlying Excel formulas used to derive all calculated parameters, which vary slightly by country and crop.

**TABLE 1:** Description of phases and parameters used in calculation flow diagram.

Phase	Parameter ID	Variable name	Variable explanation	Main data source
1. Seed availability	1A	Biofortified (BF) seed produced	Commercial BF seed produced in the country	HarvestPlus database
	1B	Total area harvested	Total area harvested (BF & NBF seed)	FAOSTAT [21]
	1C	Seed rate	Quantity of seed planted per hectare	B-R: BINA [22] P-W: Journal, Agribusiness Pakistan [23,24] N-M: IITA [25] N-C: IITA [26]
	1D	Total quantity of seed used	Total quantity of BF & NBF seed used	Calculated
	1E	Total seed produced	Total quantity of BF & NBF seed produced	B-R: MoA [27] P-W: PES [28] N-M: Journal [29] N-C: NASC [30]
	1F	Ratio of farm-saved seed/total seed	Ratio of the farm-saved seed to the total seed produced	Calculated
	1G	Farm-saved BF seed used	Total farm-saved BF seed planted	Calculated
	1H	Total BF seed used	Total quantity of biofortified seed planted	Calculated
2. Production of biofortified crop	2A	Seed rate	Quantity of seed required to plant 1 hectare	See 1C
	2B	Area planted with BF crop	Total area planted with the BF crop	Calculated
	2C	Yield for BF crop	Mean quantity of harvested crop per hectare	HarvestPlus monitoring surveys
	2D	Production of the BF crop	Total quantity of the BF crop harvested	Calculated
	2E	Farm-saved seed	Harvested grain allocated for use as seeds in the next season	FAOSTAT [31]
	2F	Animal feed	Portion allocated for animal feed	FAOSTAT [31]
	2G	Postharvest losses	Losses during processing, transportation, and storage	FAOSTAT [31]
	2H	Edible portion of the BF crop available for human consumption	Quantity of the edible BF crop available for human consumption	Calculated
3. Consumption on-farm	3A	Intake from BF crop (on-farm)	Annual per capita intake from BF crop (on-farm)	HarvestPlus monitoring surveys P-W: FAOSTAT
	3B	Farming households	Number of farming households planting the BF crop	HarvestPlus household growing model
	3C	Household size	Mean farming household size	HarvestPlus monitoring surveys P-W: Census [32] B-R: HIES [33]
	3D	Farming population	Number of people living in households producing the BF crop	Calculated
	3E	On-farm consumption	Quantity of the BF crop consumed on-farm	Calculated
4. Consumption off-farm	4A	Off-farm consumption	Quantity of the BF crop consumed by people buying from the market	Calculated
	4B	Intake from BF crop (off-farm)	Annual per capita intake from BF crop (off-farm)	FAOSTAT (assuming 100% or 50% of national mean intake coming from BF crop)
	4C	Population consuming off-farm	Number of people consuming BF crop off-farm	Calculated
5. Reach and coverage	5A	Reach	Number of individuals consuming BF crop	Calculated
	5B	Population of the country	Total population of the country	World Bank
	5C	Coverage	Proportion of the national population covered with BF crop	Calculated

Abbreviations: BF, biofortified; BINA, Bangladesh Institute of Nuclear Agriculture; B-R, Bangladesh Zinc Rice; IITA, International Institute of Tropical Agriculture; MoA, Ministry of Agriculture; NASC, The National Agricultural Seeds Council; NBF, nonbiofortified; N-C, Nigeria Vitamin A Cassava; N-M, Nigeria Vitamin A Maize; PES, Pakistan Economic Survey; P-W, Pakistan Zinc Wheat.

### Seed availability (phase 1)

The objective of phase 1 is to estimate the total quantity of biofortified seed planted in a given year. This is the sum of the annual quantity of biofortified seed acquired by farmers from seed vendors (agro-dealers and community-based seed multipliers), public seed distribution programs, and the annual quantity of “farm-saved seed” (FSS)—part of the harvested grain that is kept by farmers from the previous harvest to be used as seed in the subsequent season. The quantities of biofortified seed produced and distributed presented in this manuscript were obtained via censuses of all seed producers and vendors conducted by HarvestPlus. To collect these data, HarvestPlus staff interviewed all the producers of certified and quality-declared biofortified seed in Bangladesh, Pakistan, and Nigeria for the quantity of seed that was produced and distributed in a specific year (2023, in this case).

Unlike the commercially produced seed, the estimation of the FSS parameter requires multiple data points and back calculations, including the total area that is planted with the crop (biofortified and non-biofortified), the seed rate, and the total quantity of seed that is commercially produced. We use the total area *harvested* as a conservative proxy (total area harvested is considered a conservative proxy for area planted because adverse climatic conditions, pests, and diseases can cause farmers to harvest crops from fewer hectares than initially planted) for the total area *planted*, and by multiplying the area harvested with the seed rate, we obtained the total quantity of seed used. The difference between this quantity and the quantity of seed commercially produced results in the total quantity of seed that was saved from the last harvest as FSS. The proportion of FSS to the total seed used is then applied to calculate the quantity of FSS for the biofortified variety, as no differences in this practice of saving seeds for the next planting season are expected between non-biofortified and biofortified varieties. Estimates for the total area harvested with the crop were drawn from the FAOSTAT “Crops and livestock products” database, which takes into account annual weather- and pest-related losses [21]. The seed rate for wheat in Pakistan [24], rice in Bangladesh [22], and maize [25] and cassava [26] in Nigeria were estimated from national agricultural research agencies. Estimates for the total seeds produced came from the Ministry of Agriculture in Bangladesh [27], from the Pakistan Economic Survey in Pakistan [28], and from scientific publications for Nigeria [29,30].

Finally, the total quantity of biofortified seed used is then calculated by adding the quantity of the estimated FSS to the quantity of commercial biofortified seed produced and distributed in 2023 (Supplementary Material A for country-specific calculations).

### Production of biofortified crop (phase 2)

The objective of phase 2 is to estimate the quantity of the harvested biofortified crop that was available for human consumption in 2023. As a first step, the area (ha) planted with the biofortified crop is calculated by dividing the quantity of biofortified seed available for planting (parameter from phase 1) by the country- and crop-specific seed rate.

Subsequently, the total quantity harvested is estimated by multiplying the area planted with biofortified varieties by the country-specific mean yield for the biofortified crop, which was calculated from farmer monitoring surveys conducted by HarvestPlus in 2023 for the 4 crops with the exception of cassava where no survey was conducted in 2023. For cassava, we therefore calculated the mean yield from the 2022 survey and adjusted the yield for 2023 based on yield figures for 2022 and 2023 in the FAOSTAT “Crops and livestock products” database [FAOSTAT data (when accessed in May 2025) reported cassava yields (MT/ha) of 6.0145 in 2022 and 6.3459 in 2023, which equates to a 5.5% increase in the yield. This increased yield was applied to the 8.2 MT/ha result found in the HarvestPlus’ 2022 farmer monitoring survey (8.2 × 1.055 = 8.65 MT/ha)] [21]. Using year-specific after-harvesting farmer reported yield figures ensures that weather- or pest-related losses are considered.

As a next step, the edible quantity available for human consumption is calculated by subtracting the postharvest losses, the proportion allocated for feeding animals and FSS earmarked for the next planting season, from the quantity harvested. Farmers acquire biofortified seed using a variety of channels including direct cash purchase from seed producers or their agro-dealers, free seed through free seed distribution programs by government and development partners, from fellow farmers as gifts or paid for seed, or farmers using their FSS from harvested biofortified crops. Postharvest losses are those that occur during harvesting, threshing, cleaning, drying, transportation, and storage. Data from the FAOSTAT “Food balances” database [31] were used to determine the mean country- and crop-specific postharvest losses, quantities used for feeding animals and seed saved for the next season for the 4 crops in our study.

### On-farm consumption (phase 3)

The objectives of phase 3 are to estimate *1*) the number of individuals in farming households that consumed the biofortified crop in 2023, and *2*) the quantity (MT) of the biofortified food consumed by the farming households in 2023.

As a first step, the number of households growing the biofortified crop is multiplied by the mean household size to estimate the number of on-farm consumers. The number of households growing the biofortified crop was generated using an excel-based model that uses the number of farming households that acquire biofortified seed, the farmer-to-farmer diffusion, and attrition in each year—the farmer-to-farmer diffusion and attrition factors are derived from surveys carried out by HarvestPlus. The number of farming households for the 4 crops was estimated using data on seed sales by seed companies and seed distribution registers for public seed distribution programs by development partners that promote biofortified crops; calculations for the 4 crops are provided in Supplementary Material B. In countries that sell biofortified seed through agro-dealer networks, the number of farming households is estimated using the total quantity of seed sold and the number of seed packs sold for each seed pack size category. In this phase, the mean household size was drawn from farmer monitoring surveys conducted by HarvestPlus, which surveys farmers in locales where the majority of the conventional and biofortified crops are grown. As such, data from farmer monitoring surveys were considered more accurate than national- or regional-level household size estimates from secondary sources. In Nigeria, we used survey data from 2023 for maize and from 2018 for cassava, as more recent cassava surveys did not collect data on household size. In Bangladesh, we used data for the mean rural household size from the 2022 household income and expenditure survey [33] due to an implausibly low household size reported by the 2023 HarvestPlus farmer monitoring survey. For Pakistan, we used the mean rural household size from the 2023 census [32] because we found the data from the farmer monitoring surveys implausible.

Subsequently, the quantity of the biofortified crop consumed on-farm is calculated by multiplying the number of individuals in households farming the biofortified crop by the per capita consumption of the biofortified crop. The median per capita consumption of the biofortified variety was estimated from farmer-level monitoring surveys conducted by HarvestPlus in Bangladesh and Nigeria. In these monitoring surveys, farmers indicated the quantity of the biofortified crop allocated for consumption for the household. In Pakistan, the farmer-level monitoring data resulted in implausible consumption estimates and were thus not considered of sufficient quality for use in our calculations. We used the per capita intake from FAOSTAT instead, assuming that this applies to biofortified wheat too [31].

### Off-farm consumption (phase 4)

The objectives of phase 4 are to estimate *1*) the quantity of the biofortified crop consumed by people who obtained biofortified food from the market in 2023, and *2*) the number of individuals in households that obtain these foods from the market who consumed the biofortified food (off-farm consumption) in 2023.

The total quantity of biofortified food on the market and consumed by people who did not grow it is calculated by subtracting the quantity of biofortified food consumed on-farm (phase 3) from the total quantity that is available for human consumption (phase 2). This is the amount of the biofortified crop that is sold on the market.

To estimate the population that could consume the biofortified food obtained from the market, we used 2 consumption scenarios, both of which use the national per capita consumption of the 4 crops—taken from the FAOSTAT “Food balances” database [31]—as the basis for the calculation. In the first consumption scenario, we assumed “full replacement,” meaning 100% of consumers’ annual intake of the food crop would come from biofortified varieties . In the second scenario, we assumed “half replacement,” where 50% of consumers’ annual intake of a food crop would come from biofortified varieties. By dividing the amount of the specific biofortified food that is available on the market by the 2 scenarios of per capita consumption of the biofortified crop, we present a range of 2 estimates (based on the 2 scenarios) of the population consuming the biofortified crop off-farm.

### Reach and coverage (phase 5)

The objectives of phase 5 are to estimate *1*) the reach and *2*) the coverage of each biofortified food. The reach for a biofortified food is the sum of individuals consuming the biofortified food on-farm (phase 3) and those that consume it by purchasing from the market (phase 4). By using the 2 different scenarios in phase 4, we produce 2 estimates of the number of people reached by each crop in each country. Once the reach is calculated, the coverage can be estimated by dividing the number of people reached by a country’s total population. For our study, the population figures were drawn from World Bank estimates for 2023 [34].

## Results

The source parameters as well as the calculated parameters for all 4 crops in the 3 countries are shown in Table 2.

**TABLE 2:** Reach and coverage of biofortified crops and input parameters used for calculation.

Phases and indicators	Bangladesh	Pakistan	Nigeria	
	Rice	Wheat	Maize	Cassava^1^
Seed availability				
1A-Biofortified seed produced (MT or bundles)	7244	179,486	13,282	4,845,831
1B-Total area planted (ha)	11,641,645	9,032,688	5,700,000	9,878,773
1C-Seed rate (kg or bundles/ha)	30	120	20	60
1D-Total quantity of seed (BF&NBF) used (MT or bundles)	349,249	1,083,923	114,000	592,726,380
1E-Total seed produced (MT or bundles)	146,708	511,379^1^	60,000	79,820,000
1F-Ratio farm-saved seed/total seed	1.4	1.1	0.9	6.0
1G-Farm-saved seed available (MT or bundles)	10,001	200,954	11,954	31,138,282
1H-Total biofortified seed used (MT or bundles)	17,245	380,440	25,236	35,984,113
Production of biofortified crop				
2A-Seed rate (kg or bundles/ha)	30	120	20	60
2B-Area planted with biofortified crop (ha)	574,829	3,170,332	1,261,790	599,735
2C-Yield (MT/ha)	5.6	3.5	1.5	8.7
2D-Production of the biofortified crop (MT)	3,219,045	11,096,161	1,892,685	5,188,801
2E-Farm-saved seed (%)	2	4	1	0
2F-Animal feed (%)	13	2	39	33
2G-Postharvest losses (%)	4	4	5	8
2H-BF crop available for consumption (MT)	2,640,819	9,980,420	1,095,556	3,196,561
Consumption on-farm				
3A-Annual per capita intake from biofortified crop by farming households (kg)	187	103	20	89
3B-Farming households growing the biofortified crop (#)	2,552,319	3,276,066	1,962,832	2,236,726
3C-Household size of farming households growing the biofortified crop (#)	2.8	6.3	9.1	5.6
3D-Farming population consuming the biofortified crop (#)	7,146,493	20,639,216	17,861,771	12,525,666
3E-On-farm consumption of the biofortified crop (MT)	1,336,394	2,125,839	357,235	1,114,784
Consumption off-farm				
4A-Off-farm consumption of the biofortified crop (MT)	1,304,425	7,854,581	738,321	2,081,777
4B.1-Annual per capita intake from BF crop (off-farm), 100% from BF crop (kg)	247	103	31	162
4B.2-Annual per capita intake from BF crop (off-farm), 50% from BF crop (kg)	123	52	15	81
4C.1-Population consuming off-farm 100% from BF crop (#)	5,283,212	76,258,065	23,893,871	12,882,281
4C.2-Population consuming off-farm 50% from BF crop (#)	10,566,423	152,516,129	47,787,742	25,764,563
Reach and coverage				
5A.1-Reach (100% from BF crop off-farm) (#)	12,429,705	96,897,280	41,755,642	25,407,947
5A.2-Reach (50% from BF crop off-farm) (#)	17,712,916	173,155,345	65,649,513	38,290,228
5B-Population of the country (#)	171,466,990	247,504,495	227,882,945	227,882,945
5C.1-Coverage (100% from BF crop off-farm) (%)	7	39	18	11
5C.2-Coverage (50% from BF crop off-farm) (%)	10	70	29	17

Abbreviations*:* BF, biofortified; NBF, nonbiofortified.

1 Quantity of seeds measured in bundles.

In Bangladesh, in 2023, 17,245 MT of zinc biofortified rice seed was estimated to be available for planting. This resulted in the production of an estimated 3.2 million MT of biofortified rice grain, of which around 2.6 million MT was available for human consumption after deducting for seed saved for the next planting season, postharvest losses, and grain used to feed animals. Around 50% of the rice available for human consumption was consumed on-farm, with the remaining portion entering the market (1.3 million MT) and being available for those who buy from the market. We estimated that 7.1 million people consumed biofortified rice that they grew on their own farms. The 2 scenarios used to estimate the number of people who consumed biofortified rice obtained from the market (100% or 50% of annual per capita intake covered by foods made from biofortified rice) resulted in a range of 5.3–10.6 million people that could be reached with biofortified rice. In total (on-farm and off-farm consumption), between 12.4 and 17.7 million people could be reached with biofortified rice. This would correspond to a coverage of 7%–10% of the Bangladeshi population.

In Pakistan, in 2023, 380,440 MT of zinc biofortified wheat seed was estimated to be available for planting resulting in a production of an estimated 11.1 million MT of biofortified wheat grain of which 10.0 million MT were available for human consumption. Around one-fifth of the produced biofortified wheat was directly consumed on-farm (2.1 million MT), and 7.9 million MT were consumed by people obtaining it from the market. Our calculations estimate that around 20.6 million people consumed biofortified wheat that they grew on their own farms. The estimated number of people who consumed biofortified wheat food obtained from the market (100% or 50% of annual per capita intake covered by foods made from biofortified wheat) resulted in a range of 76.3–152.5 million people that could be reached with biofortified wheat. In total (on-farm and off-farm consumption), our estimates show that between 96.9 and 173.2 million people could have consumed biofortified wheat food in 2023. This would correspond to a coverage of 39%–70% of the population in Pakistan consuming biofortified wheat.

In Nigeria, in 2023, 25,236 MT of vitamin A biofortified maize seed and 35,984,113 bundles of vitamin A biofortified cassava were estimated to be available for planting. This resulted in a production of around 1.9 million MT of biofortified maize of which 1.1 million MT were available for human consumption after considering deductions for FSS for the next planting season, postharvest losses and grain used to feed animals. Around one-third of the grain produced was consumed on-farm (357,235 MT). The remaining part, 738,321 MT, was consumed by people who obtained it from the market. Of the 5.2 million MT of biofortified cassava produced, 3.2 MT was available for human consumption after considering losses. Around one-third (1.1 million MT) of biofortified cassava was estimated to be consumed by people who grew it on their own farms, with a remaining 2.1 million MT being available on the market for those who buy from the market. The 2 scenarios used to estimate the number of people who consumed biofortified food by purchasing from the market (100% or 50% of annual per capita intake covered by foods made from the biofortified crops) resulted in a range of 23.9–47.8 million people consuming biofortified maize and between 12.9 and 25.8 million people consuming biofortified cassava food buying from the market. The total number of people reached, including on-farm and off-farm consumption, resulted in a range of 41.8–65.6 million people that could be reached by biofortified maize and between 25.4 and 38.3 million people that could be reached by biofortified cassava. This would correspond to a coverage of 18%–29% of the Nigerian population for biofortified maize and 11%–17% for biofortified cassava.

## Discussion

In this manuscript, we describe a comprehensive approach to calculate the reach and coverage of 4 biofortified foods in 3 countries. This was accomplished without the use of nationally representative survey data, which is often not available or, when available, may be an unreliable source of reach/coverage for biofortified foods. By utilizing secondary data sources and data from small-scale monitoring surveys conducted by HarvestPlus, our approach enables managers of biofortification programs and policymakers to routinely estimate the reach and coverage of biofortified foods.

Estimating the reach and coverage of biofortification programs in this manner has clear programmatic benefits. First, program managers can regularly report reach and coverage estimates to national stakeholders to inform public health nutrition programming. This is particularly important as the quantity of biofortified crops produced in most countries is increasing. Second, this method can be used to estimate the reach and coverage of biofortified crops with invisible traits, which cannot be readily measured by coverage surveys, as survey respondents are not always able to determine if the food they consume originated from a biofortified or a nonbiofortified crop [17]. This methodology is also novel and cost-effective, enabling stakeholders to track program performance and estimate the contribution of biofortification to the reduction of micronutrient deficiencies.

### Comparison with previous estimates

As estimating the reach and coverage of biofortified foods is a nascent endeavor, we only identified previous analyses from Nigeria with which to compare our results. A 2022 paper by Birol et al. [35] described the evolution of Nigeria’s biofortification program and estimated that 13 million individuals consumed biofortified maize and cassava in 2021. The authors noted that this estimate was conservative as *1*) it did not include off-farm consumption, and *2*) it assumed that 25% of individuals from farming households consumed both biofortified maize and cassava. Using similar assumptions, our estimated on-farm reach from Table 2 (parameter 3D) would be 22.8 million [on-farm reach estimates of 17.9 million for biofortified maize and 12.5 million for biofortified cassava and 25% overlap (i.e., (17.9 m + 12.5 m) × (1 – 0.25)]. The difference observed between our on-farm estimates and those from Birol et al. is explained by both the increased number of farmers cultivating biofortified maize and cassava in 2023 compared with 2021, and the differences in the mean household size. Birol et al. assumed an mean *national* household size of ∼ 5 individuals (personal communication), whereas we utilized the mean household size from farmer monitoring surveys in different demographic regions of Nigeria (9.1 for maize and 5.6 for cassava). We deemed household size data from the farmer monitoring survey superior to national means, as the household sizes reported by farmers account for regional differences in household sizes. To illustrate, orange maize is mostly grown in Northern Nigeria, where surveys of maize farming households have previously reported household sizes of 8 members in Jigawa state [36] and 11 members in Katsina state [37]. Nonetheless, the difference between Birol et al.’s on-farm reach estimate and our reach estimate is relatively small. This underscores the importance of estimating off-farm reach of biofortified food. In Nigeria, we estimate that off-farm reach of biofortified maize is ≈1.3–2.6 times higher than on-farm reach, and the off-farm reach of biofortified cassava similar or double on-farm reach.

Our results can also be compared with cross-sectional survey results produced as part of Nigeria’s 2021 National Food Consumption and Micronutrient Survey [38]. This survey reported that 13.5% and 3.4% of women of reproductive age consumed biofortified maize or cassava in the past 30 d, respectively. The survey’s coverage estimate for biofortified maize is slightly lower than our 2023 coverage estimates of 18%–29%, which could be explained by the increased production of biofortified maize between 2021 and 2023. Similarly, the survey’s coverage estimate for biofortified cassava is slightly lower than our 2023 coverage estimates of 11%–17%. Although the 2021 survey asked participants if they consumed the “yellow (biofortified) cassava, or any food products made from it, in the previous 30 d,” it is possible that the respondents underreported their consumption of biofortified cassava. This is because gari from biofortified cassava and white gari with palm oil added are both yellow and visually indistinguishable [39], which potentially cause respondents to misidentify their consumption of biofortified cassava. In contrast to the 2021 national survey, which reported a coverage of 1.7% in South-West zone, a small survey, also conducted in 2021, by Akinsola et al. [40] from Oyo state—also in Nigeria’s South-West zone—estimated that 37.5% of “cassava product consuming households” consumed biofortified cassava. The estimate by Akinsola et al. is higher than our biofortified cassava coverage estimate, which may be due to the fact that the study by Akinsola et al. excluded households that did not consume cassava products and that production of cassava is higher in the Southern part of Nigeria [41].

### Parameter variability

Our reach and coverage estimates are mainly based on primary data from small surveys and secondary data from a variety of sources if primary data were not available. Because the parameter values published by different researchers and institutions vary, the calculated reach and coverage values change depending on the parameter value that is used. In the calculations presented in this paper, we validated the sources and judged our assumptions to be reasonable. To illustrate, the household size of farming households is used to estimate the on-farm reach and subsequently calculate the quantity (in metric tons) of the biofortified crop that is consumed on-farm and sold on the market. As described previously, we used household size values taken from farmer-level monitoring surveys conducted by HarvestPlus because we found this was a better data source than public databases that provide the mean household size at the national, urban/rural, or regional levels. Future users of this method should clearly state/cite the data source for the household size and other parameters they used in the calculation.

### On-farm consumption per capita

In farmer monitoring surveys that are regularly conducted by HarvestPlus, farmers are asked whether they have planted specific biofortified crops, and if yes, farmers are subsequently asked the quantity they harvested and to estimate the quantity they allocate for their own consumption. Using consumption data from such surveys comes with challenges, as the distribution of the consumption data is usually skewed. Because of the positive skewness of the distribution of consumption values from farmer monitoring surveys, we used per capita *medians* calculated to estimate the quantity of the biofortified crop that is retained for own consumption for all farm households that grow the biofortified crop. This was done because preliminary analyses using mean per capita values (data not shown) resulted in some calculations where the estimated quantity of biofortified foods consumed on-farm exceeded the estimated harvest.

In Bangladesh, the median per capita intake on-farm consumption used in our calculation is 187 kg/person/y. This is approximately three-quarters of the per capita consumption (247 kg/person/y) reported by FAOSTAT [31]. In Nigeria, per capita median on-farm consumption was 20 kg/person/y for biofortified maize and 89 kg/person/y for cassava. These estimates are approximately two-thirds and one-half the per capita consumption figures reported by FAOSTAT [31]. For both Bangladesh and Nigeria, these consumption estimates are plausible as the farmer monitoring surveys indicated that nearly all farmers grow both biofortified and non-biofortified varieties of each crop; hence, they will meet part of the food crop requirement from the non-biofortified food crop.

In Pakistan, we considered that the data for per capita wheat consumption from farmer monitoring surveys were too high and implausible to use. The mean annual per capita median consumption from farmer monitoring surveys in 2023 and 2024 was 259 kg. This would translate into an mean daily per capita median consumption of ∼710 g (259 × 1000 ÷ 365), which as considerably higher than other estimates and deemed implausible by the authors. For this reason, we used the FAOSTAT per capita intake for the on-farm consumption (i.e., 103 kg/person/y) and the mean rural household size from the 2023 census [32]. Due to the large-scale adoption and high levels of production of zinc wheat in Pakistan [42,43], we can plausibly assume that people in farms that grow zinc wheat consume exclusively biofortified wheat, and as such, using 100% of the FAOSTAT per capita food supply value is plausible.

### Off-farm consumption estimates affect reach and coverage

When estimating the off-farm reach, we used 2 consumption estimates—100% of the per capita annual consumption from FAOSTAT and 50% (half) of the same per capita annual consumption from FAOSTAT. This produces a range in the reach and coverage estimates. This range is needed as there is currently scant data describing the consumption of biofortified food by nonagricultural households. The authors are only aware of 1 such survey—Nigeria’s 2021 National Food Consumption and Micronutrient Survey—that estimated the coverage of biofortified foods nationally via a household-based survey where there were no restrictions on household enrollment. However, as noted previously, this survey may have underestimated the off-farm coverage of biofortified cassava, as the main end product (i.e., yellow gari) cannot be distinguished visually from conventional gari prepared with palm oil. Moreover, the high cost of such surveys discourages carrying them out frequently, hence the need for alternative methods.

We also tested a different method to estimate the annual consumption of the biofortified crop by people buying biofortified foods from the market. Specifically, we calculated the proportion of biofortified seeds to total seeds acquired by farmers for a specific crop and multiplied this same proportion by per capita annual consumption figures obtained by FAOSTAT. This approach assumed full mixing of the biofortified crop with the non-biofortified crop before it appears on the market, which does not account for the fact that biofortified crops with visible traits are typically sold and processed separately from conventional varieties, nor local differences in consumption patterns. Using this proportion for the 4 crops resulted in higher reach and coverage estimates, as the assumption of complete mixing produces a lower per capita intake than the 2 scenarios used in our calculations. The biofortified seed-to-total seed ratio was 5% for zinc rice in Bangladesh, 35% for zinc wheat in Pakistan, and 22% for vitamin A maize in Nigeria. This would increase the reach and coverage for rice in Bangladesh to 59.2 million people and 35% of the total population, for wheat in Pakistan to 237.9 million people and 96%, and for maize in Nigeria to 125.8 million people and 55%. This approach did not work for vitamin A cassava in Nigeria, where the proportion of biofortified cassava stems to total cassava stems was 6%, which, when inserted into our calculation, produced a reach that was higher than the total population of Nigeria. Due to this, we decided not to use this method.

Understanding the variation in the consumption of these foods by different geographies would be needed to produce accurate estimates of the reach and coverage of a national population. In the absence of these data, estimating the off-farm reach using 2 consumption values is a viable approach, as it enables the estimation of the reach that could be obtained assuming that individuals off-farm fully or partially substituted conventional crop varieties with the biofortified alternative.

The off-farm consumption of biofortified foods with invisible traits is also challenging as there is currently little information detailing to what extent biofortified foods are mixed with conventional varieties. Indeed, the mixing of biofortified foods with conventional varieties by grain aggregators has been reported [44]. Although being part of national value chains is key to the sustainable distribution of biofortified foods, the mixing of biofortified crops and non-biofortified varieties could suggest that nascent biofortification programs will have a high off-farm reach/coverage but will have minimal health impact due to the “dilution” of the biofortified food. However, to the contrary, and from a food system’s perspective, what matters may be the quantity of micronutrients added to the food system, not whether it is eaten as pure or mixed. Indeed, health impact could be achieved as the share of biofortified crops to the total food crop increases. Additional work estimating the contribution of biofortified crops to the micronutrients in a food system will be necessary, as will a detailed analysis of the value chain processes linked to biofortified foods.

### Further research and validation

Many of the parameters used in our calculation come from secondary data sources. Although these secondary data sources are useful and reliable, additional research activities should be conducted to collect primary data for various parameters to help improve the accuracy of the reach and coverage figures. First, because our calculation currently uses an off-farm consumption range, rapid (e.g., phone-based) surveys can be used to identify regions in a country that have or do not have access to biofortified foods with visible traits. Although this could be only done for biofortified foods with visible traits, it could nonetheless help refine the off-farm consumption value included in the equation. Importantly, although these data could improve the performance of this calculation, it would not produce a viable national estimate of the reach and coverage of biofortified foods. Second, for biofortified crops with invisible traits, better data would be required on the extent to which producers and aggregators mix them with conventional varieties. Our calculation assumes that crops with invisible traits (e.g., biofortified wheat and rice) are mixed with conventional varieties only on the farm; however, it is important that a value chain analysis that “follows” biofortified crops through the value chain—from the farm to distribution points to the households— be used to estimate the extent of mixing that occurs, which in turn can be used to refine the off-farm consumption value.

These metrics will likely be country- and crop-specific, as they will need to account for off-farm consumption of biofortified crops with visible traits and/or the proportion of a crop with invisible traits that is biofortified.

### Strengths and weaknesses

Our methodology provides a useful starting point for estimating the reach and coverage of biofortified foods. A key strength of our method is the use of agricultural production and consumption estimates. Although this method requires the extensive use of secondary data sources, it produces reach and coverage estimates with primary data that can be easily and rapidly collected. Furthermore, our method can estimate the off-farm reach and coverage of biofortified foods with invisible traits, which is something that traditional coverage surveys cannot accomplish [17]. Its cost-effectiveness compared with the household surveys makes it such a valuable tool that can be used to provide useful estimates by policymakers. The methodology considers country-crop–specific nuances, including sociocultural consumption assumptions, ensuring that there is no 1-size-fits-all approach to all crops.

A key weakness of our method is that it requires careful consideration of parameter values, as these can vary considerably between datasets (see above). As such, the user of this method must use his/her judgment when inserting parameter values into the calculation and must justify the inclusion of parameter values from 1 source over another. As such, we strongly recommend that future calculations describe in detail all sources used and describe instances where the parameter values from ≥2 sources varied considerably. Another weakness is that our calculation does not account for exports and imports of biofortified foods. Although the exportation and importation of the country-crop combinations we examined is minimal, the export and import of biofortified crops may need to be accounted for in some instances.

Overall, our approach enables managers of biofortification programs to produce plausible and reproducible reach and coverage estimates using a combination of primary and secondary data for biofortified crops with visible and nonvisible traits. We estimate that in 2023, biofortified food crops in Bangladesh, Pakistan, and Nigeria were consumed by 177 and 293 million people, assuming a replacement rate of 100% and 50%, respectively. This estimate does not account for potential overlap in the consumption of biofortified cassava and maize in Nigeria. Between 13 and 16 million people consumed biofortified rice in Bangladesh, 97 and 173 million people consumed biofortified wheat in Pakistan, and 42 and 66 million people and 25 and 38 million people consumed biofortified maize and biofortified cassava in Nigeria, respectively.

## Author contributions

The authors’ responsibilities were as follows — RA, BM: designed the research and primary responsibility for the final content; RA, RW, BM, JPW, EB: conducted the research; RA, RW, BM: analyzed the data; RA, RW, BM, JPW: wrote the paper; and all authors: contributed to the manuscript revisions and read and approved the final manuscript.

## Data availability

The underlying data used for this manuscript, including results from unpublished HarvestPlus postharvest farmer monitoring surveys, can be made available on reasonable request. Data sharing requests should be sent to the corresponding author at B.Mudyahoto@cgiar.org.

## Funding

This study was funded by International Food Policy Research Institute (IFPRI) via a grant award (2025X035.GRO) to GroundWork.

## Conflict of interest

We hereby declare that none of the authors have any conflict of interest regarding this review, and that the manuscript or portions of it have not been published and are not under consideration for publication in any other journal and have not been posted on the internet. This article was funded by HarvestPlus. Richard Alioma, Bho Mudyahoto, Wolfgang Pfeiffer, Munawar Hussain, and Erick Boy are affiliated with HarvestPlus, which is a program of the Innovations, Policy and Scaling Unit of the International Food Policy Research Institute (IFPRI).
